# Application of the dual-factor model of mental health among Chinese new generation of migrant workers

**DOI:** 10.1186/s40359-021-00693-5

**Published:** 2021-11-30

**Authors:** Qingji Zhang, Jinglin Lu, Peng Quan

**Affiliations:** 1 School of Marxism, Dongguan University of Technology, Dongguan, 523808 China; 2 Research Center for Quality of Life and Applied Psychology, Key Laboratory for Quality of Life and Psychological Assessment and Intervention, Guangdong Medical University, Dongguan, 523808 China

**Keywords:** Dual-factor model, Positive mental health, Psychopathology, New generation migrant workers

## Abstract

**Background:**

Traditional models of mental health focus on psychopathological symptoms. In contrast, the dual-factor model of mental health integrates positive mental health and psychopathology into a mental health continuum, which is an adaptation and complement to the traditional mental health research paradigm. The new generation of migrant workers is an important part of current Chinese society. Their identity has created a sense of loneliness, rootlessness, and alienation. This paper validates the applicability of the dual-factor model of mental health among new generation migrant workers in China.

**Methods:**

In this study, 600 new generation migrant workers were recruited and tested on the symptom checklist 90, satisfaction with life scale, perceived stress scale, employee engagement inventory. Descriptive statistics and ANOVA were performed, the differences between the unidimensional model and dual-factor model were also tested.

**Results:**

The results showed that the dual-factor model of the mental health approach had better construct validity than the unidimensional model. And four subgroups could be significantly discriminated by the dual-factor model: mentally healthy (58.45%), vulnerable (30.87%), symptomatic but content (3.11%), and troubled (7.57%). Compared to the other three groups, workers who were mentally healthy showed higher perceived work values and lower perceived work stress.

**Conclusions:**

The study suggests that a dual-factor model of mental health can be applied to new generation migrant workers in China, with positive mental health and psychopathology being important predictors of mental health.

## Background

Chinese new generation of migrant workers are those born after 1980, who still hold rural household registration (Hukou) and have been engaged in non-agricultural employment or non-local employment in the local area for more than six months. According to the “2020 Migrant Worker Monitoring Survey Report” released by National Bureau of Statistics of China, there will be 285.6 million migrant workers in China in 2020 [1]. The new generation of migrant workers has become an important part of Chinese society.

In recent years, China’s economic growth has improved the well-being of most residents [2]. However, the impact of economic growth on life satisfaction varies across social groups. Especially, life satisfaction of migrant workers is lower than both urban and rural residents [3]. For migrant workers, the higher their socioeconomic status in the city, the higher their life satisfaction. However, when objective socio-economic status such as income and occupation of migrant workers generally improve, their influence on life satisfaction gradually weakens, while subjective social status, as an important complementary dimension of socio-economic status, can more significantly and positively predict life satisfaction.

The new generation of migrant workers are characterized by low literacy, low educational attainment, and low income. Due to the different social environment and education level, the new generation of migrant workers has obvious differences from the older generation of migrant workers in terms of development space, identity and sense of belonging [4]. The new generation of migrant workers have a strong competitive edge in employment, higher expectations of life, and a stronger sense of defending their rights. In addition to emphasizing real material benefits such as wages, the new generation of migrant workers have a strong demand for social benefits such as employment, education, medical care, retirement and housing [5]. Moreover, they demand a broader space for development and pursue self-actualization more actively. On the other hand, they are also facing an identity crisis. They live and work in the city, hoping to be fully integrated into the city, but are not accepted by the city because China’s current household registration system does not allow them to enjoy the social benefits of city residents. As a result, they are in the non-rural and non-urban margins of both systems. This awkward situation of their identity has created a sense of loneliness, rootlessness and alienation. Studies have showed that Chinese new generation migrant workers had significantly more psychological symptoms than others [6, 7]. The psychological health of young migrant workers was declining from 1995 to 2011, with both hostility and anxiety being particularly prominent [8].

The traditional concept of mental health is based on a unidimensional model that assumes that mental health is the absence of psychopathology. However, with the development of positive psychology, academics have begun to question this traditional conceptual classification criterion of mental health because it places more emphasis on the negative indicators and ignores the positive indicators [9].The absence of psychopathology is not the same as the presence of positive mental health. Positive mental health and psychopathology are not poles of one dimension, but form two negatively correlated but independent factors [10]. Thus, traditional assessment methods may overestimate or underestimate mental health. Specifically, the mental health status of individuals who do not meet the diagnostic criteria for mental illness but still feel distressed is overestimated, and the ability of individuals with mental illness but high levels of well-being to recover themselves is underestimated [11].

Therefore, researchers have proposed a dual-factor model of mental health, which includes two main indicators of positive mental health and psychopathology [12, 13]. The model classifies mental health into four types: mentally healthy, vulnerable, symptomatic but content, and troubled. The dual-factor model presents a complete operational concept of mental health, thus providing new idea and framework for mental health interventions. The value of this theory has been validated by many studies [14]. Currently, the four-group classification of the dual-factor model has been widely used in the assessment of mental health of elementary, secondary, and college students in a Western cultural context [9, 11, 15]. Research supports the idea that mental health is comprised of separate dimensions of well-being and psychopathology [9]. Psychopathology may coexist with high level of well-being, and conversely, the absence of psychopathology and low well-being may coexist [13]. Some students were mentally healthy. Vulnerable students did not exhibit clinical psychopathology, but reported a relatively poor well-being. Students who were symptomatic but content, did not appear to suffer from distress due to their positive perceptions of life. The troubled students had significant mental health issues and reported a poor sense of well-being [11]. Several empirical studies have found that the four subgroups classified according to the dual-factor model show clinically significant differences. The four subgroups differed significantly in locus of control, attention problems, alcohol abuse, hope, and gratitude [15]. The four subgroups also differed greatly in life satisfaction, sense of life, flourishing level, and depression [16]. The intervention plan for different mental health subgroups will be individualized, including reducing mental illness, and simultaneously working to improve well-being.

However, research on the dual-factor model of mental health among adult populations in Chinese culture remains scarce. This study first examines whether the dual-factor mental health model is applicable to the new generation of Chinese migrant workers. Further, high levels of mental health are believed to help motivate individuals and maintain their employee engagement [17]. And psychological well-being is negatively related to perceived work stress [18]. Therefore, the present study also wished to investigate whether there were differences in the clinical manifestation of mental health (employee engagement and perceived stress) among the four subgroups grouped based on the dual-factor model.

## Methods

### Participants

The survey site is a typical city with a high concentration of new generation migrant workers in China, namely the famous manufacturing base, Dongguan. The new generation of migrant workers in the electronics and machinery industries were selected as the survey respondents. A total of 600 questionnaires were distributed with a return rate of 85.8% (N = 515). The demographic characteristics of the current sample are shown in Table 1.

**Table 1:** Demographic characteristics of the current sample

	Frequency	Percentage (%)		Frequency	Percentage (%)
Gender			Marital status		
Male	48	9.32	Single	223	43.30
Female	465	90.29	Married	286	55.53
Unknown	2	0.39	Unknown	6	1.17
Age			Children		
16–22	110	21.36	0	337	65.44
21–23	112	21.75	1	132	25.63
23–26	142	27.57	2	38	7.38
26–30	98	19.03	≥ 3	2	0.39
30–39	43	8.35	Unknown	6	1.17
≥ 40	4	0.78	Sexual partnership		
Unknown	6	1.17	None	187	36.31
			Any	309	60.00
			Unknown	19	3.69
Education					
Junior high school or below	328	63.69	Housing status		
High school	170	33.01	Buying	83	16.12
College	8	1.55	Renting	415	80.58
Bachelor and above	1	0.19	Unknown	17	3.30
Unknown	8	1.55			
Religion			Native place		
None	451	87.57	Local	12	2.33
Any	45	8.74	Other city	425	82.52
Unknown	19	3.69	Unknown	78	15.15

### Measures

#### Symptom Checklist 90 (SCL-90)

SCL-90 is a 90-item inventory, which comprises the following nine domains: somatization (12 items), obsessive compulsive (10 items), interpersonal sensitivity (9 items), depression (13 items), anxiety (10 items), hostility (6 items), phobic anxiety (7 items), paranoid ideation (6 items), psychoticism (10 items), and 7 items were not attributed to any other domain [19]. The Chinese version of the SCL-90 is used to measure current psychological symptom status, which is used in the vast majority of studies on migrant workers [20]. And in this study the Cronbach’s α of above-mentioned ten dimensions successively was 0.786, 0.617, 0.773, 0.588, 0.725, 0.810, 0.624, 0.651, 0.692, 0.714. The McDonald’s ω was 0.792, 0.623, 0.775, 0.588, 0.732, 0.818, 0.627, 0.627, 0.707, 0.726.

#### Satisfaction with Life Scale (SWLS)

The SWLS is a short 5-item unidimensional instrument designed to measure an individual’s overall subjective satisfaction with life [21]. The SWLS is a 7-point Likert scale. The item is like “In most ways my life is close to my ideal”. Higher scores indicate a higher level of satisfaction with life. The Chinese version of the SWLS demonstrated high internal consistency, reliability, and validity [22]. And in the current study, the Cronbach’s α was 0.686. The McDonald’s ω was 0.689.

#### Perceived Stress Scale (PSS)

The PSS is a 14-item instrument, which is rated on a 5-point Likert scale [23]. A higher total score on the PSS indicates a higher level of perceived stress. The PSS consists a negative subscale (7 items) and a positive subscale (7 items). The item is like “In the last month, how often have you been upset because of something that happened unexpectedly?” The Chinese version of PSS demonstrated adequate reliability and validity [24]. In the current study, the Cronbach’s α of the total scale was 0.697. The McDonald’s ω was 0.699.

#### Employee engagement inventory (EEI)

The instrument consists of 17 items with a 5-point Likert scale, including the following three domains: work engagement, organizational identity, perceived work values [25]. The higher the score, the higher the level of employee engagement. Work engagement has 7 items that describe how engaged employees are at work, e.g., “When I’m working, all I can think about is my work”. Organizational identity has 5 items that describe employees’ identification with the organization, e.g., “I am proud to tell others that I am part of this company”. Perceived work values has 5 items that describe whether or not employees feel that they are valued in their work, e.g., “I find the work I do to be full of meaning and value”. The Cronbach’s α of above-mentioned three domains and total scale was 0.411, 0.636, 0.875. The McDonald’s ω was 0.422, 0.639, 0.879. The domain of work engagement was excluded from the formal analysis due to low reliability. In the current study, we used only two domains (organizational identity, perceived work values).

### Procedure

This survey was conducted as part of a comprehensive survey of employee mental health in two labor-intensive industries in Dongguan, Guangdong, China. The data were collected in October 2015. The survey was authorized by the companies, and volunteered participants. The research team recruited 12 survey assistants to collect data. First, the research team promoted the survey through a range of advertising techniques (i.e. flyers, posters, and social media). Then, participants who volunteered for the survey were asked to meet at a designated location and assigned to a quiet room to complete the questionnaire. The Institutional Review Board of Dongguan University of Technology approved the research protocol and conducted overall study oversight and monitoring. Informed consent was obtained from each participant prior to data collection. In order to ensure the anonymity of the participants, we do not collect their personal information (e.g. name, phone number, address). All data were stored in password-protected files that can only be used by the research team for the purpose of data analysis. Based on the judgment of psychologists in the research team, participants were referred for psychotherapy/counseling if needed. All participants received a gift of approximately $1 as compensation.

### Statistical analysis

Quantitative analyses were conducted in SPSS 25.0 and AMOS 25.0. Descriptive statistics (mean, standard deviation) were calculated for all variables. The reliability related to internal consistency is measured by the *Cronbach’s α* and *McDonald’s ω.* Statistical significance level was set to 0.05. To explore the relationship between psychiatric symptoms and well-being, a structural equation modeling technique was used to assess the unidimensional and dual-factor model. Specifically, two competing structural equation models were tested to explore the relationship between psychiatric symptoms and well-being. *McDonald’s omega* was calculated using a SPSS macro [26]. Model fit is assessed by a series of goodness-of-fit indicators, including the *χ*^2^ value and the degrees of freedom (*χ2/df*), Goodness-of-Fit Index (*GFI*), adjusted GFI (A*GFI*), normed fit index (*NFI*), comparative fit index (*CFI*), Tucker–Lewis index (*TLI*) and root mean square residual (*RMR*), root mean square error of approximation (*RMSEA*). The values of *GFI, AGFI, NFI, CFI, TLI* are close to 0.95, the value of *RMR* is close to 0.05, and the cutoff value of *RMSEA* is close to 0.05, which indicates a good fit between the hypothesized model and the observed data. The sensitivity of the dual-factor model was assessed by the ability to identify different subgroups (ANOVA). In addition, post hoc analyses were conducted to determine which groups differed significantly from each other.

## Results

### Common-method bias test

Harman’s one-factor test was conducted for all items in the four questionnaires. The results showed a *KMO* value of 0.883. A total of 36 factors with eigenvalues greater than 1 explained 70.05% of the variance; the first factor explained 20.04% of the variance, well below the 40% threshold [27]. This suggests that the bias effect of the common-method was not significant in this study.

### Validation of the dual-factor model of mental health with psychiatric symptoms and well-being as assessment indicators

The structural equation model was constructed using a combination of questionnaire items. In this study, Model 1 was a unidimensional model with only one latent variable representing overall mental health; psychiatric symptoms represented a negative factor with a negative loading on the latent variable, while well-being represented a positive factor with a positive loading on the latent variable. Model 2 is a dual-factor model in which psychiatric symptoms and subjective well-being each represent two different latent variables. These two potential variables are related, but independent of each other. As shown in Table 2, model 2 outperformed model 1 for various goodness of fit indicators, especially *χ*^*2*^*/df*, *AGFI*, *RMR*, and *RMSEA*. thus, the dual-factor model of mental health was better than the unidimensional model.

**Table 2:** The goodness of fit of the unidimensional and dual-factor model

	*χ*^*2*^	*df*	*χ*^*2*^*/df*	*GFI*	*AGFI*	*NFI*	*CFI*	*TLI*	*RMR*	*RMSEA*
Model 1	301.48	90	3.35	0.92	0.90	0.93	0.95	0.96	0.18	0.07
Model 2	127.95	86	1.49	0.97	0.96	0.97	0.99	0.97	0.05	0.03

### Mental health status of new-generation migrant workers

Using psychiatric symptoms as a negative indicator and well-being as a positive indicator, we divided the mental health status of new generation migrant workers into four subgroups. As shown in Table 3, referring to the approach described by Eklund et al. [15], participants who scored ≥ 3 on any of the factors of SCL-90 (i.e., the factor caused “moderate” or “high” distress to the participant) were defined as having high psychiatric symptoms. Participants scoring < 3 were defined as having low psychotic symptoms. Participants who scored ≥ 4 on the SWLS (i.e., were “mostly satisfied” or above with their current state of life) were defined as having high mental health, whereas participants who scored < 4 were defined as having low mental health. The results showed that 58.45% (N = 301) were in the positive mental health group, 30.87% (N = 159) were in the vulnerable group, 3.11% (N = 16) were in the symptomatic but content group, and 7.57% (N = 39) were in the troubled group. We examined whether the four subgroups differed in demographic characteristics. There were no differences in gender, education, religion, sexual partnership, or housing status (*p*s > 0.1). There were differences in age, marital status, children, and native place (*p*s < 0.05).

**Table 3:** Groups yielded from dual-factor model of mental health

Level of SCL-90	Level of SWLS	
	Low	High
High	Troubled	Symptomatic but content
	≥ 3 on any of the factors of SCL-90	≥ 3 on any of the factors of SCL-90
	< 4 on the SWLS	≥ 4 on the SWLS
Low	Vulnerable	Positive mental health
	< 3 on any of the factors of SCL-90	< 3 on any of the factors of SCL-90
	< 4 on the SWLS	≥ 4 on the SWLS

### The group differences among new generation migrant workers

We also examined whether the four subgroups differed in their organizational identity, perceived work values, and perceived work stress. We assessed group differences in the scores of EEI domains and PSS total score using one-way ANOVA.

As shown in Table 4, no difference between groups was found in the domain of organizational identity. In terms of perceived work values, post hoc comparisons using LSD correction showed that the two groups characterized by high level of satisfaction with life (positive mental health and symptomatic but content) did not have significantly difference (*p* = 0.86) and both were higher than the group characterized by low well-being (vulnerable, troubled) (*ps* < 0.05). For perceived work stress total score, positive mental health group had significantly lower stress scores than other three subgroups (symptomatic but content, vulnerable, troubled) (*ps* < 0.01). The vulnerable group also had lower stress level than the troubled group (*p* = 0.01). And there was no difference between the other groups.

**Table 4:** Means and standard deviations of EEI and PSS scores

	Positive mental health	Vulnerable	Symptomatic but content	Troubled	*F*
Organizational identity	4.06 ± 0.64	3.85 ± 1.11	4.16 ± 0.53	3.93 ± 1.22	2.44
Perceived work values	3.86 ± 0.66	3.35 ± 0.82	3.83 ± 0.70	3.35 ± 0.94	19.20***
PSS Total score	1.67 ± 0.43	1.94 ± 0.46	2.01 ± 0.31	2.15 ± 0.50	21.09***

***p < 0.001, **p < 0.01, *p < 0.05

## Discussion

### Validation of a dual-factor model using psychiatric symptoms and well-being as assessment indicators

In this study, psychiatric symptoms were used as a negative indicator and well-being as a positive indicator in order to construct structural equation models to test the goodness of fit of the unidimensional model and the dual-factor model of mental health. Our results showed that the dual-factor model had a better goodness of fit, which is consistent with previous studies [11, 15, 28]. This suggests that the dual-factor model of mental health is applicable to the new generation of migrant workers. The traditional concept of mental health, which defines mental health based on the absence of mental illness, is a one-sided view. Psychiatric symptoms and well-being are not two ends of the same dimension, but two related but independent constructs.

### Mental health status of new-generation migrant workers

This study found that 58.45% of the new generation of migrant workers were mentally healthy, the percentage of those with positive mental health was the highest among the four groups. This is consistent with the results of previous studies on adolescents [11, 15, 28].

It is worth to notice that 30.87% of the new generation migrant workers belonged to the vulnerable group, which meant that they did not have psychiatric symptoms but were less satisfied with their lives. The reason may lie in the dual status of the new generation of migrant workers. They have rural Hukou and live in towns, but they cannot enjoy the benefits of towns. Compared with urban people, the new generation of migrant workers have a lower socio-economic status. According to the traditional concept of mental health, they are out of the scope of concern. However, the mental state of vulnerable groups leads them to be more prone to reduced sense of value, purpose, and efficacy, family conflicts, low work efficiency, and chronic diseases. The older generation of migrant workers alleviate their sense of relative deprivation by comparing themselves with farmers in their hometowns, while the new generation of migrant workers prefer to choose urban residents as their reference group, even though this makes them feel a stronger sense of relative deprivation [29]. Therefore, more preventive efforts should be made to address the mental health of such populations.

According to our findings, as policy makers, officials can enhance the new generation of migrant workers’ identity and urban integration in order to improve their life satisfaction and enhance their sense of well-being. For example, they can work on social security, children’s schooling and other rights and interests that new generation migrant workers should have.

### Characteristics of perceived work values and perceived work stress among new generation migrant workers with different mental health status

Grouped according to the dual-factor model, the four groups of new generation migrant workers differed significantly in terms of perceived work values and perceived stress. This result is consistent with the findings of a previous study [10]. Further analysis revealed that the positive mental health group had higher perceived work values than vulnerable group. This may indicate that increasing the life satisfaction of new generation migrant workers may also increase their employment stability and reduce frequent quitting and job hopping or long-term unemployment [30]. In terms of perceived work stress, positive mental health, vulnerable, symptomatic but content, and troubled group had sequentially higher stress perceptions, which may indicate a protective effect of mental health on work stress.

### Limitations and future research directions

The purpose of this study was to explore the applicability of the dual-factor model of mental health to new generation migrant workers in China. The limitations of this study are mainly as follows. First, the study population from the single center was relatively small. Therefore, future research needs to expand the breadth and depth of the survey. Second, this study is a cross-sectional investigation and cannot test the longitudinal effects of the dual-factor model on individual growth. Therefore, future research should examine the meaning and value of this model from a longitudinal perspective. Finally, this study is a quantitative investigation. Therefore, future research should investigate the differences of the four groups from a qualitative perspective.

## Conclusion

The dual-factor model of mental health is applicable to the new generation of migrant workers in the Chinese manufacturing industry. Psychiatric symptoms and well-being are two important dimensions of mental health. The proportion of new generation migrant workers in the four subgroups was similar to that of the youth population in a previous study. New generation migrant workers with mentally healthy had the highest levels of perceived work values and the lowest levels of perceived work stress.

## Data Availability

The datasets generated for this study are available on reasonable request from the corresponding author.

## Abbreviations

SCL-90: Symptom Checklist 90
SWLS: Satisfaction with Life Scale
PSS: Perceived Stress Scale
EEI: Employee engagement inventory
